# M. Andral on Cholera

**Published:** 1831

**Authors:** 


### XXXVIII.

### M. ANDRAL ON CHOLERA.

THIS eminent pathologist has lately been amusing a Parisian audience with a disquisition on a disease which he has never seen — with whose *pathology* he acknowledges himself ignorant—whose causes he has been unable to discover — but whose treatment is laid down with boldness — while he ridicules the methodus medendi employed by those who have seen the disease in all its direst and most deadly forms. He tells us, what every body knew before, that, "on opening the bodies of those who sink under cholera, there can be discovered no notable alteration either in the intestinal canal or its appendages." Hence he thinks cholera should be classed with the *enteralgic* rather than with the *enteritic* diseases. The treatment which *he* (who never saw the disease) would recommend, is free bleeding among the young and vigorous—external irritation — and plenty of laudanum internally. Now all these means have been employed, and with little success. But because the English have found by repeated experience, that calomel is useful in allaying the gastric and intestinal irritability in this dreadful disease, M. Andral seizes the opportunity of ridiculing the English practice. "I cannot account," says he, "for the prostrate veneration which English physicians pay to this metallic drug. I can only compare them to those poor Indians who, faithful to their ancient creed, persist, with words of mystic import, in plunging their sick into the charmed waters of the Ganges." Wonderful infatuation! Strange that a people who have carried every improvement, in art and science, immeasurably beyond the French, should yet be so stupid as not to see that every disease is *gastro-enterite*, and that gum-water, leeches, and a glyster-pipe are all that a physician can want or wish in this or any other climate! Finally, says M. Andral, "physicians in India dose their cholerous patients with a draught composed of brandy, rum, pimento, pepper, camphor, &c. which, I must confess is little better than a kind of *sauve qui peut* remedy." MED. GAZ.

Now this sagacious *pathologist*, who appears in this instance at least, to be no great physician, tells us that cholera is *enteralgic* rather than enteritic; and if so, why should not spices and stimulants be allowed, as well as laudanum? But the fact is, he knows nothing whatever of the nature, cause, or treatment of a disease on which he has descanted largely, in order to cast reflections on English physicians.

